# Does the Macro-Temporal Pattern of Road Traffic Noise Affect Noise Annoyance and Cognitive Performance?

**DOI:** 10.3390/ijerph19074255

**Published:** 2022-04-02

**Authors:** Beat Schäffer, Armin Taghipour, Jean Marc Wunderli, Mark Brink, Lél Bartha, Sabine J. Schlittmeier

**Affiliations:** 1 Empa, Swiss Federal Laboratories for Materials Science and Technology, 8600 Dübendorf, Switzerland; armin.taghipour@hslu.ch (A.T.); jean-marc.wunderli@empa.ch (J.M.W.); lel.bartha@gmail.com (L.B.); 2 School of Engineering and Architecture, Lucerne University of Applied Sciences and Arts, 6048 Horw, Switzerland; 3 Federal Office for the Environment (FOEN), 3003 Bern, Switzerland; mark.brink@bafu.admin.ch; 4 Faculty of Engineering, University of Regina, Regina, SK S4S 0A2, Canada; 5 Institute of Psychology, RWTH Aachen University, 52056 Aachen, Germany; sabine.schlittmeier@psych.rwth-aachen.de

**Keywords:** road traffic noise, macro-temporal pattern, noise indicator, noise annoyance, cognitive performance, Stroop task, listening experiment

## Abstract

Noise annoyance is usually estimated based on time-averaged noise metrics. However, such metrics ignore other potentially important acoustic characteristics, in particular the macro-temporal pattern of sounds as constituted by quiet periods (noise breaks). Little is known to date about its effect on noise annoyance and cognitive performance, e.g., during work. This study investigated how the macro-temporal pattern of road traffic noise affects short-term noise annoyance and cognitive performance in an attention-based task. In two laboratory experiments, participants worked on the Stroop task, in which performance relies predominantly on attentional functions, while being exposed to different road traffic noise scenarios. These were systematically varied in macro-temporal pattern regarding break duration and distribution (regular, irregular), and played back with moderate *L*_Aeq_ of 42–45 dB(A). Noise annoyance ratings were collected after each scenario. Annoyance was found to vary with the macro-temporal pattern: It decreased with increasing total duration of quiet periods. Further, shorter but more regular breaks were somewhat less annoying than longer but irregular breaks. Since Stroop task performance did not systematically vary with different noise scenarios, differences in annoyance are not moderated by experiencing worsened performance but can be attributed to differences in the macro-temporal pattern of road traffic noise.

## 1. Introduction

Noise annoyance is one of the most important negative health-related effects of environmental noise [1,2]. For annoyance, exposure-response relationships are typically based on time-averaged metrics, such as the A-weighted equivalent continuous sound pressure level (*L*_Aeq_), the day-night level (*L*_dn_), or the day-evening-night level (*L*_den_) [3,4,5]. However, while such noise metrics have proven to be strong predictors of annoyance (e.g., [4,6]), they ignore other potentially important acoustical and non-acoustical characteristics of a noise situation, in particular the macro-temporal pattern (e.g., [7,8,9,10,11]). The objective of our study therefore was to elucidate the link between the macro-temporal pattern of road traffic noise and annoyance on the one hand, and cognitive performance on the other hand, especially as the latter might moderate annoyance ratings, and because evidence of noise effects on cognitive performance is still scarce [12]. Note that the term “road traffic noise” is used throughout this paper to refer to either road traffic induced “noise” or “sound”. The term “road traffic noise” is very common (e.g., [6]). However, strictly speaking, sound and noise are not the same. Sound refers to the physical quantity sound pressure from which acoustical metrics can be derived with calculations or measurements, while noise refers to unwanted sound entailing negative effects on humans (e.g., [6]). As a consequence, studies on negative effects rather refer to noise, while soundscape studies focusing on potentially positive effects refer to sound (e.g., [13]).

Road traffic noise and its effects on annoyance and cognitive performance becomes increasingly important as urbanization is progressing. While less than 34% of the global population lived in urban regions in 1960, this number rose to more than 56% globally in 2020 (and to ~74% in Europe) [14]. This growth of urban areas goes hand in hand with an increase in noise pollution, in particular due to road traffic. Accordingly, some 113 million Europeans were estimated to be exposed to road traffic noise *L*_den_ of 55 dB or more in 2017 [15], of which more than 72% lived in urban areas. Increasing road traffic noise calls for effective countermeasures (noise control and mitigation) to be considered by urban planners. They need to know which acoustic qualities and quantities they have to preserve or (re-)create in remnant or newly designed urban spaces. This, however, requires sufficiently funded knowledge on the effects of traffic noise. While much research was dedicated to noise annoyance in the past (e.g., [2]), effects on cognitive performance are less explored [16,17]. A recent systematic review of non-experimental studies on the association between transportation noise and cognitive performance found only 34 papers, which did not allow for a quantitative meta-analysis and were exclusively dedicated to child populations [12]. Thus, studies on mutual effects of road traffic noise on annoyance and cognitive performance of adults are desirable.

The macro-temporal pattern of noise and its effect on **noise annoyance** may be described with different indicators. The number of dominant events, typically defined relative to a threshold (e.g., Number above Threshold, NAT [18]), has been reported to be a promising predictor of annoyance [9,19,20], and also the maximum sound pressure level (*L*_A,max_) is occasionally used for the same purpose [21]. Besides, one may use statistical levels, namely, *L*_10_, *L*_50_ and *L*_90_, to describe rare events, average noise levels and background noise [22,23], respectively, or differences between statistical levels to define fluctuation and/or emergence [24]. Further, quietness was suggested as an additional predictor for (reduced) noise annoyance [7,10]. Finally, the eventfulness of noise situations, expressed as intermittency ratio [11], was proposed as an additional indicator for annoyance. Literature indeed suggests annoyance to be associated with such indicators for the macro-temporal pattern of noise. One study found reduced annoyance in highly intermittent road traffic noise situations with only a small number of vehicles per hour [5], which might be the consequence of phases of relative quietness between events, lasting two or more minutes on average. Several other studies emphasized the need to consider quiet periods (i.e., noise breaks) in the assessment of noise impact on public health [8,25,26,27,28,29]. They suggested that not only the total length of noise breaks, but also their distribution and individual duration could be important [8,25,27], as longer breaks (in total and individually) might mitigate annoyance [8,9,10,25,27]. Here, a minimum duration of noise breaks seemed necessary to be noticeable and effective [25,27,28,29], which should last one minute, called “a while” (“eine Weile” in German) [25], or three minutes [27,28,29]. Calm periods were also found in [30] to reduce annoyance, while their pattern (regular or irregular) did not have a significant effect. However, with 0.25–1.65 s, the noise breaks were quite short. Thus, the macro-temporal pattern may be decisive for annoyance, but literature on this aspect is still quite scarce.

In addition to annoyance, the macro-temporal pattern of road traffic noise may also affect **cognitive performance**. In everyday life and at work, cognitively demanding tasks often have to be achieved in the presence of background noise. Consequently, the detrimental effects of task-irrelevant sound on cognitive performances have been explored in a multitude of basic cognitive psychological studies (see, e.g., [31,32,33]). However, whereas quite some research focused on chronic effects of road traffic noise on children’s cognitive performance [12], surprisingly little evidence is available on acute effects on cognitive performance of adults (e.g., [17,34,35,36,37]). With regard to the macro-temporal pattern of road traffic noise as constituted by the duration and distribution of noise breaks, the effect on attentional functions is of particular interest. This is because unexpected, salient changes in the acoustic background cause the distraction of the attentional focus from the task to the background sound, so that controlled task-related processes are interrupted. This attentional capture and resulting drop in cognitive performance is known as the “deviance effect” [38]. It occurs because our auditory-cognitive system constantly monitors the acoustic background, at least to a certain extent, even when we are concentrating on a given visual cognitive task unrelated to the noise. In fact, a certain distractibility is an important prerequisite for human survival in potentially threatening environments. However, when focusing on a cognitive task, road traffic noise is arguably irrelevant in all respects. Nonetheless, its macro-temporal pattern may cause attentional capture, in particular the transitions from noisy to quiet periods and back, and/or irregular noise breaks as unanticipated changes in the auditory background. Yet while the length and distribution of noise breaks appear to affect noise annoyance, their effects on attentional capture have not been studied to our knowledge. Since subjective annoyance ratings and cognitive task performance do not necessarily go hand in hand, it is not possible to infer from noise effects on annoyance to cognitive performance effects [39,40,41]. Thus, both effect dimensions should be studied for a comprehensive evaluation of road traffic noise and its macro-temporal pattern, even more so as impacts on cognitive performance might moderate noise annoyance, and as mutual effects of road traffic noise have hardly been studied so far. For example, one might notice that his/her own performance is reduced under road traffic noise, and this is then expressed in a higher subjective annoyance rating.

The objective of the present study therefore was to investigate the effects of the macro-temporal pattern of different road traffic scenarios on noise annoyance and objective performance indicators of attentional functions by means of psychoacoustic laboratory experiments.

## 2. Methodological Approach

**Figure 1:**
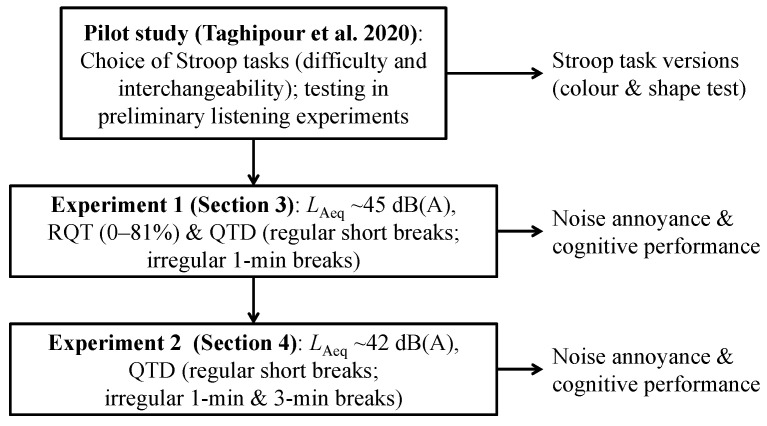
Study design: Pilot study to this paper by Taghipour et al. [44] to identify suitable Stroop task versions, experiment 1 on the association of noise annoyance and cognitive performance with relative quiet time (RQT) and quiet time distribution (QTD), and experiment 2 on the association with QTD. Details are given in [44] (pilot study) as well as in Section 3 and Section 4 (experiments 1 and 2).

In this study, two experiments were conducted to investigate the effects of the two independent macro-temporal pattern variables “relative quiet time” and “quiet time distribution” (cf. Section 2.3) on short-term noise annoyance and cognitive performance in a task which predominantly relies on attentional functions: the Stroop task [42]. Experiment 1 investigated the individual and combined effects of the two variables, while experiment 2 focused on the effect of quiet time distribution in more detail. Two different versions of the Stroop task, derived from the colour test [42] and shape test [43], were used (Section 2.2). The latter were identified as suitable in a pilot study to this paper [44], where (i) the difficulty of Stroop tasks necessary for the framework of our study was assessed, (ii) interchangeable Stroop tasks were identified, and (iii) the chosen tasks were applied in a preliminary listening experiment to test their feasibility. The pilot study is described in detail in [44]. Figure 1 gives an overview of the workflow of the experiments.

In the following, Section 2.1 introduces the experimental concept of our study, Section 2.2 presents the Stroop tasks, and Section 2.3 the indicators used to quantify the macro-temporal pattern of the road traffic noise scenarios. Section 3 then documents experiment 1 and Section 4 experiment 2. Section 5 discusses the results, before Section 6 gives the major conclusions to our study.

### 2.1. Experimental Concept: Unfocussed Listening Experiments

In two experiments, subjectively perceived acute noise annoyance reactions (so called “short-term annoyance” [45,46] or “psychoacoustic annoyance” [47]) to road traffic noise scenarios with different macro-temporal pattern were investigated under laboratory conditions. Each scenario was several minutes long (4.5 min in experiment 1 and 10 min in experiment 2) and comprised a number of single car pass-by events.

The listening experiments were designed as “unfocused listening experiments” (e.g., [48,49]), where the participants’ primary focus was not on the noise scenarios but on a cognitive task (see below). While focused listening experiments are widely used in studies where participants attentively listen to and rate acoustic stimuli of relatively short duration (usually <1 min; e.g., [45,48]), unfocused experiments are typically performed for subjective assessment of noise scenarios with considerably longer durations as used here (several minutes or hours; e.g., [17,49,50]). Furthermore, the latter experimental set-ups allow both measuring the effects of sound on cognitive performance and to collect subjective annoyance (or other) ratings of the sound situations.

**Figure 2:**
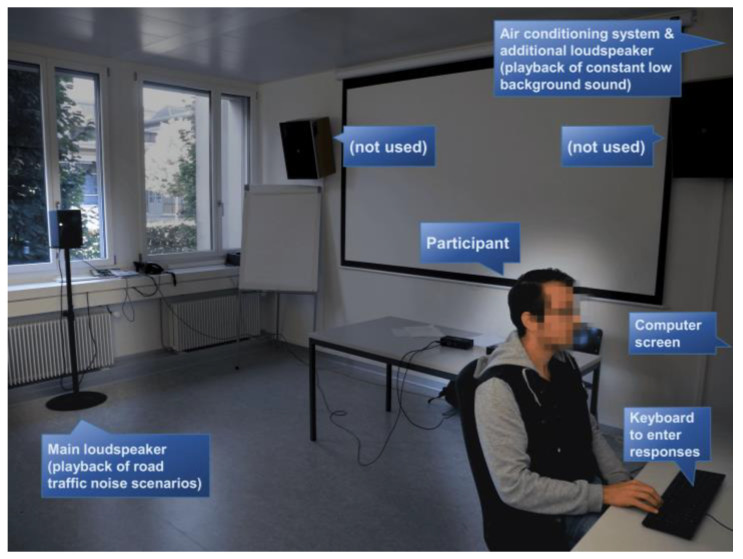
Photography of the laboratory setup (office environment at the Empa) used for the listening experiments. The loudspeaker positioned at the (closed) window mocked the road traffic noise scenarios at an open window, while the participant at a desk performed the visually presented cognitive task. The loudspeakers on the wall (left and right of the picture and indicated with “not used”) were not used in the current experiments. For details on the air conditioning system and the additional loudspeaker see Section 3.1.

In the present study, the participants conducted a visually presented cognitive task, while road traffic noise scenarios were played back. The participants’ primary focus was thus on the cognitive task and not on the noise scenarios. However, at the end of each noise scenario, the participants rated their noise annoyance. As laboratory setup, an office environment was chosen where an open window was simulated from which the road traffic noise would enter the office (Figure 2). To that aim, a loudspeaker playing back road traffic noise scenarios was placed in front of the closed window. For the experiments, moderate exposure scenarios with *L*_Aeq_ of 42–45 dB(A) were chosen, which are representative values for an office environment. The daytime limit value (impact threshold) for road traffic noise of 60 dB outdoors in residential zones according to Swiss legislation [51] and a sound level attenuation during transmission from the outside to the inside of some −15 dB for tilted windows [52,53] approximately result in the above indoor *L*_Aeq_. Likewise, a road traffic noise *L*_den_ of 53 dB according to the recommendation of WHO [6], corresponding to a daytime *L*_Aeq_ of ~51 dB(A) [54], and a sound level attenuation during transmission from the outside to the inside of some −10 dB for open windows [53] lead to similar values. Besides the actual noise scenarios, constant low background sound was played back with an additional loudspeaker (cf. Section 3.1).

The experiments were approved by the ethics committee of Empa (approval CMI 2019-224 of 30 October 2019). They followed general guidelines such as [55,56] and were conducted similarly to previous experiments by the authors (e.g., [21,45]).

### 2.2. Stroop Task Versions for Unfocussed Listening Experiments

**Figure 3:**
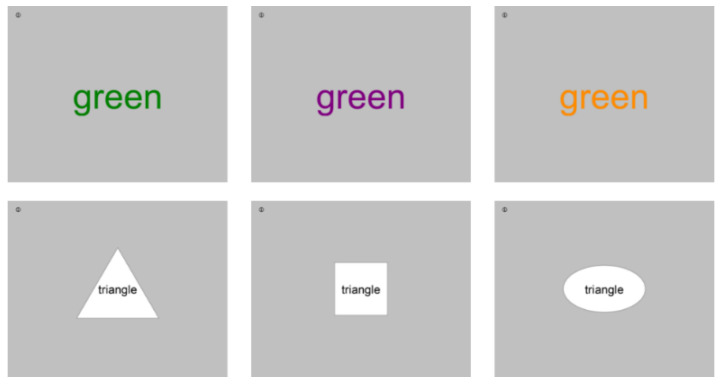
Screenshot examples of the colour version (**top**) and the shape version (**bottom**) of the Stroop task used in this study. Congruent items (the two stimulus’ attributes match) are shown left and incongruent items (stimulus’ attributes do not match) in the middle and right.

Cognitive performance was tested using different versions of the Stroop task. Details on the Stroop task are given, e.g., in [57]. In its standard version, different colour words are displayed (blue, green, red, yellow) which are either printed in the same colour as their semantic meaning (congruent item; e.g., the word “green” displayed in green colour) or in another colour (incongruent item; e.g., the word “green” displayed in blue) [42] (cf. first row of Figure 3). Participants are asked to respond to the colour in which the word is printed (in the latter example: blue) and not the word’s semantic (here: green). Reading the semantics of a word is an automated process for skilled readers, so that in the case of incongruent items the automatically activated word must be inhibited and the correct response–namely, the print colour of the word–must be specifically selected. Therefore, an increase in errors and/or response times occurs for incongruent items compared to congruent items, which is the so-called Stroop effect [42,58].

**Figure A1:**
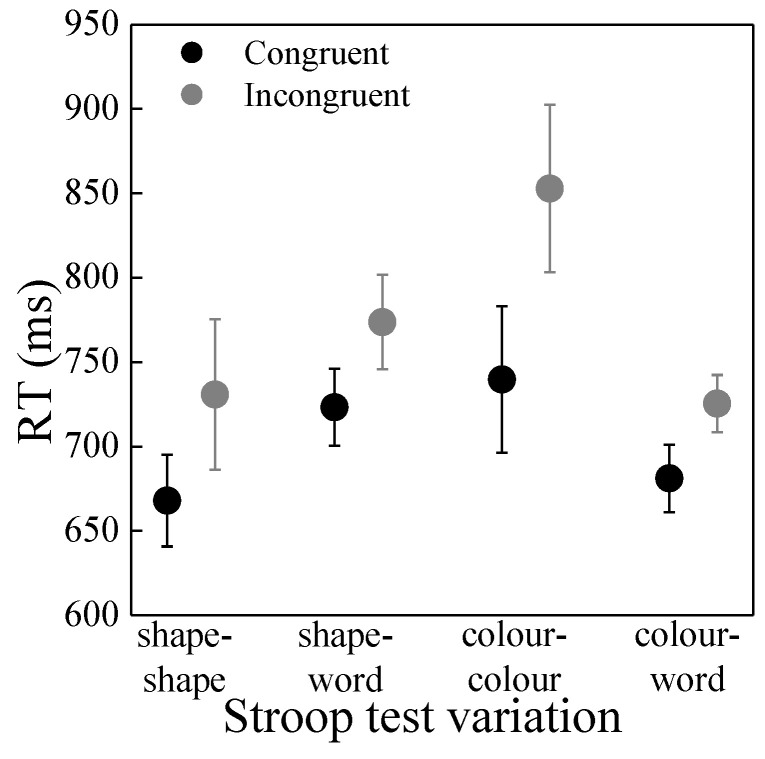
Results of a pilot experiment to this study (details see [44]): Mean reaction time (RT) with standard error bars, shown separately for congruent and incongruent trials, for four variations of the Stroop task: (i) shape test naming the shape of a geometric form with a word written within (shape-shape), (ii) shape test naming the written word within a geometric form instead of its form (shape-word), (iii) colour test naming the print colour instead of the semantics of the word (colour-colour), (iv) colour test naming the semantics instead of the print colour of the word (colour-word).

Performance in the Stroop task relies on attentional functions, namely, selective attention and inhibitory functions, so that it should be sensitive to attentional capture induced by transitions from a quiet period to road traffic noise or vice versa. As working on a large amount of look-alike items for prolonged time periods might become too tiresome, different versions of the Stroop task were used in the present study. Two versions of the Stroop task were identified in a pilot experiment to this study (details see [44]) as sufficiently equivalent with respect to difficulty, interchangeability and observability of the aforesaid Stroop effect (cf. Figure A1 in the Appendix A). The first version was a colour test where, contrary to its standard version ([42], see above), participants were asked for the semantics of the colour word (instead of its actual print colour) (cf. first row of Figure 3). The second version was a shape test (cf. [43]), where participants were asked to identify the shape of a geometric form, while a written word within specified the same or a different geometric form (cf. second row of Figure 3). Here, congruent items are those in which the semantic meaning of the word and geometric shape match (e.g., the word “rectangle” is printed in a rectangle), while these do not match for incongruent items (e.g., the word “rectangle” is printed in a circle while the latter should be named).

In addition to the above two versions of the Stroop Tasks, two variants each were used to keep the task to be processed sufficiently diverse:

- Shape test variant A: oval, square, and triangle (cf. Figure 3)
- Shape test variant B: circle, rectangular, and star
- Colour test variant A: green, orange, and purple (cf. Figure 3)
- Colour test variant B: blue, yellow, and red

The different versions/variants of the Stroop task were implemented in a listening test program in the Python-based PsychoPy software environment [59]. The individual trials were presented on a monitor screen, and responses were given by the participants on a keyboard and stored by the program.

### 2.3. Indicators for the Macro-Temporal Pattern of the Road Traffic Noise Scenarios

The following indicators were used to quantify the macro-temporal pattern of the road traffic noise situations: Number of events (*N*), Relative Quiet Time (RQT), Intermittency Ratio (*IR*), Centre of Mass Time (CMT), A-weighted and FAST-time weighted maximum sound pressure level (*L*_AFmax_), and Quiet Time Distribution (QTD). The indicators were all calculated from the road traffic noise scenarios (see below) in MATLAB Version 2019a (The MathWorks, Inc., Natick, MA, USA).

Number of events (*N*): Since this study used isolated car pass-by events mixed to prepare the scenarios (see below), the number of events, as well as the logarithm log(*N*) as sometimes used to predict annoyance (e.g., [20]), in each scenario were directly available.

Relative Quiet Time (RQT): Based on suggestions by [10], RQT is determined as the ratio of total duration of quiet periods (*T*_quiet_) to total duration of a scenario (*T*_scenario_) [26]. To that aim, *T*_quiet_ is calculated as the sum of all (individual) quiet periods and divided by *T*_scenario_ as

(1) $$\mathrm{RQT}\;\left(\%\right)=100\cdot \frac{T_{\mathrm{quiet}}}{T_{\mathrm{scenario}}}$$

Intermittency Ratio (*IR*, %): *IR* is a measure for the eventfulness of a noise scenario [11]. It expresses the proportion of the acoustical energy of all individual noise events relative to the total sound energy of a scenario as

(2) $$IR\;\left(\%\right)=100\cdot 10^{0.1\left(L_{\mathrm{Aeq},T,\mathrm{Events}}-L_{\mathrm{Aeq}}\right)},$$

where *L*_Aeq,T,Events_ is calculated from contributions of events exceeding a given threshold *K*. In contrast to other descriptors working with thresholds, the latter is not constant, but defined dynamically relative to the *L*_Aeq_ of the scenarios using

(3) $$K=L_{\mathrm{Aeq}}+C\left[\;\mathrm{dB}\right],$$

where *C* is a constant offset, set to 3 dB. *IR* ranges from 0–100%. An *IR* larger than 50% indicates that more than half of the total sound energy is due to distinct pass-by events. In situations where all events clearly emerge from background noise (e.g., at a receiver close to a railway track), *IR* gets close to 100%, while constant road traffic as observed from a receiver not too close to a motorway yields only small *IR* values. Note that while a high *IR* is a precondition for noise breaks (large RQT) to occur, it does not allow studying the effect of QTD (i.e., the temporal distribution and length of the noise breaks).

Centre of Mass Time (CMT): CMT is an indicator for quiet periods which penalizes the fragmentation of quiet periods and rewards their clustering and thus increases with longer quiet time periods [8]. It is calculated as

(4) $$\mathrm{CMT}\;\left(s\right)=\frac{\sum t_{i}{}^{2}}{\sum t_{i}},$$

where *t_i_* is the duration of the i-th (individual) quiet period in the scenario (in seconds).

Quiet Time Distribution (QTD): QTD is a categorical variable for the nature of noise breaks. Here, it discriminates between regular and irregular temporal distribution of the breaks as well as between different durations of the irregular noise breaks.

## 3. Experiment 1

In experiment 1, the individual and combined effects of the independent macro-temporal pattern indicators RQT and QTD on noise annoyance and cognitive performance in the Stroop task were investigated.

### 3.1. Audio Processing and Resulting Road Traffic Noise Scenarios

Road traffic noise scenarios (WAVE PCM format) were prepared in MATLAB Version 2019a (The MathWorks, Inc., Natick, MA, USA) from stereo recordings with a Jecklin disk setup made within a previous study [45], of individual car pass-by events which were dominated by tire/road noise. Since the laboratory setup should represent an office environment in which the road traffic noise enters through an open window, the signals were down-mixed from stereo to mono by means of crossfading. The recordings, processing, and playback was carried out at a sampling frequency of 44.1 kHz.

Road traffic noise scenarios were created from excerpts of the individual car pass-by events by mixing them together sequentially (and sometimes slightly overlapping) in time. After careful inspection of the audio files (audibly as well as based on their A-weighted and FAST-time weighted level-time histories, *L*_AF_), an average duration of 10 s was chosen for the excerpts. However, to obtain realistic sound scenarios, three excerpts, of 9, 10, and 11 s length, were cut from each signal. One of these three excerpts per event was randomly chosen for the preparation of a scenario. The excerpts were gated with raised-cosine ramps of 2 s. They were further highpass and lowpass filtered at 52 Hz and 10 kHz, respectively, to consider the limits of the loudspeaker at low frequencies and inherent recording noise at high frequencies. In total, seven scenarios, each lasting 4.5 min, were prepared for experiment 1. Additionally, two 30 s long road traffic noise scenarios were created for the participant’s familiarization period with the noise and the cognitive task at the beginning of the experimental session.

**Figure 4:**
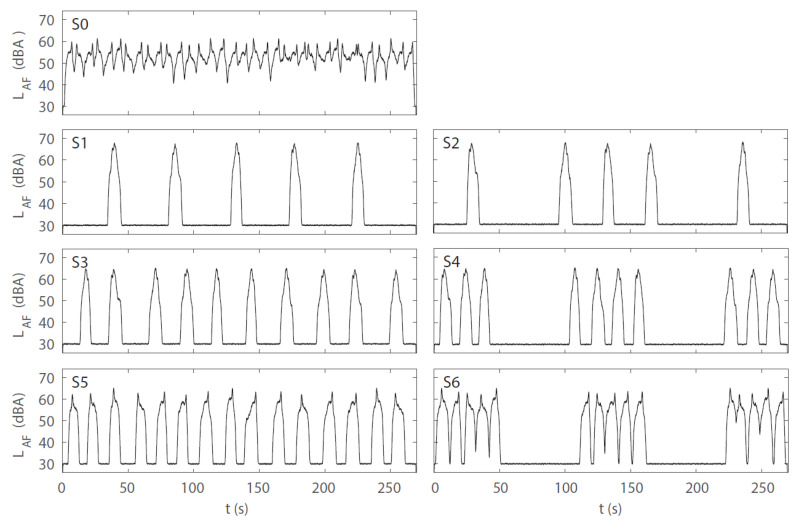
A-weighted and FAST-time weighted level-time histories (*L*_AF_) of the road traffic noise scenarios in experiment 1. S0–S6 refer to scenario 0 (reference) to 6 (cf. Table 1).

**Figure 5:**
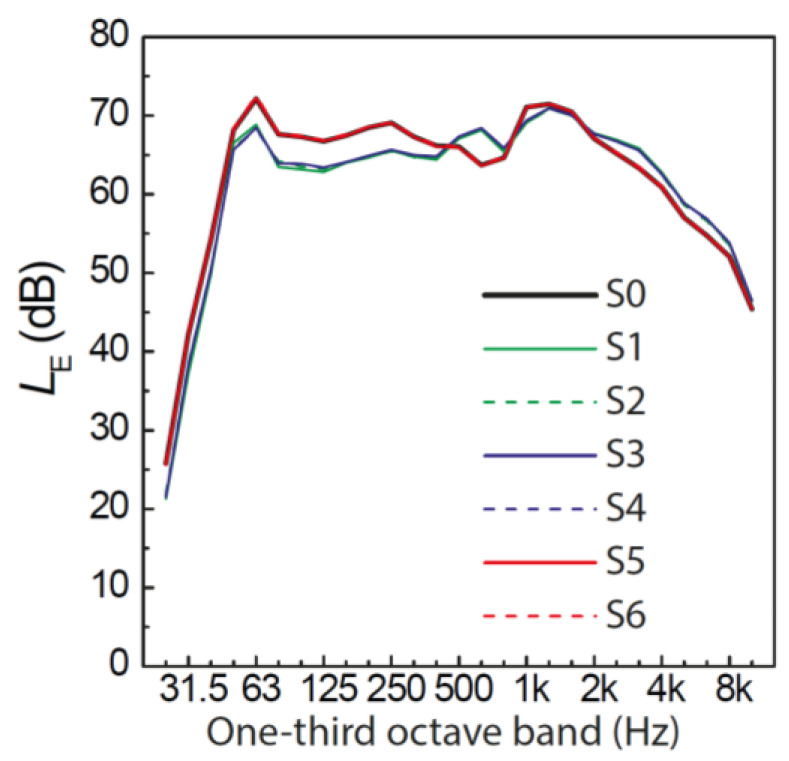
One-third octave spectra of the road traffic noise scenarios in experiment 1. S0–S6 refer to scenario 0 (reference) to 6 (cf. Table 1).

The road traffic noise scenarios covered four levels of RQT, namely, 0.0% (corresponding to 36 car pass-by events), 44.3% (15 events), 62.9% (10 events), and 81.5% (5 events). Further, two types of QTD were used for the quiet periods: either a regular distribution (referred to as “regular” in the following account) or a combination of short quiet periods and two longer (1-min) quiet periods (referred to as “irregular”). While the situation with 0.0% RQT served as a reference without quiet periods, the three levels of RQT (44.3%, 62.9%, 81.5%) were combined with the two QTD types, (total of 3 × 2 + 1 = 7 road traffic noise scenarios). All road traffic noise scenarios had the same *L*_Aeq_ of 54 dB(A) at the window (measured 50 cm away from and in front of the loudspeaker) and of 44.5 dB(A) at the participant’s ear level at the desk. As the number of car pass-by events varied between scenarios, the *L*_Aeq_ of the individual pass-by events had to be adjusted. Figure 4 shows the level-time histories of the road traffic noise scenarios, visualizing different distributions and resulting lengths of the quiet periods, and Figure 5 the corresponding one-third octave spectra, which were all very similar. Table 1 presents the indicators for the resulting macro-temporal pattern of the scenarios, and Table A1 in the Appendix A presents the correlation analysis using Spearman’s rank correlation coefficient (*r*_s_) [60] for the continuous indicators, as a measure of similarity of the indicators without an a priori assumption of a linear relation. While the *L*_AF,max_ generally decreases with increasing number of events to obtain the same overall *L*_Aeq_ for all scenarios, a few events of scenarios S5 and S6 (each encompassing 15 events) had a similar *L*_AF,max_ as the events of S3 and S4 (each encompassing 10 events), so that the *L*_AF,max_ were almost identical for those four scenarios (Table 1). N, RQT, *IR* and *L*_AF,max_ were closely correlated to each other. CMT, in contrast was not correlated to these indicators (Table A1), but was closely related to QTD, with substantially larger values for irregular than for regular distributions (Table 1). Thus, with N, *IR* and *L*_AF,max_ being closely related to RQT and CMT being closely related to QTD, the association of the macro-temporal pattern with annoyance and cognitive performance was mainly investigated with RQT and QTD (cf. Section 3.4 and Section 3.5).

Note that in addition to these road traffic noise scenarios, the participants were exposed to a constant background sound with an *L*_Aeq_ of 30 dB(A), which was a combination of filtered pink noise (played back via an additional loudspeaker) and sound from a low-level running office air conditioning system. The additional loudspeaker was located at the wall in front of and above the participant, at the same height as the running low-level office air-conditioning system, so that both sounds were received from roughly same direction and combined to one background sound source. The background sound helped masking possible low-level sounds from outside the office environment, which was not an isolated listening booth. In addition, a sign was put up during the experiments in the corridor outside the office, asking passers-by to be silent. Thus, sounds from outside the office were minimized. With the played-back background sound being constant and ~15 dB lower than the actual road traffic noise scenarios, both sound sources (sound outside the office and background sound) are negligible as a source of bias for the annoyance ratings. Also, even if the background sound within the mock office would have somewhat affected the participants’ perception and/or performance, this is something that would also be present in a real office environment.

### 3.2. Experimental Procedure

The experiments were conducted in single sessions in English. To ensure sufficient understanding of the experimental tasks, one requirement for study participation was to have good self-reported English language skills. In addition, after task instruction the participants could ask the experimenter in case of ambiguities.

Participants first answered questions about their hearing status, vision, and well-being for inclusion and exclusion criteria, which were (i) self-reported normal hearing (not hearing impaired), (ii) self-reported normal or corrected-to-normal vision (but not colour blind), (iii) legal age (18 years or older) and (iv) feeling well (not further specified). Thereafter, they read instructions on the road traffic noise scenarios, the cognitive task and the test program. To familiarize them with the two versions of the Stroop task, the two short road traffic noise scenarios were used: Participants worked on trials of the colour version of the Stroop task during the first short scenario and of the shape version during the second one. Then, data collection in the actual listening experiment started. During each noise scenario, the participant worked on trials of one version of the Stroop task for the first 135 s and then of the other version for the second 135 s. Congruent and incongruent trials were presented in random order. An overall mixing ratio of approximately 50% each was secured by the program increasing the probability of drawing either the congruent or incongruent trials after 60% of a noise scenario’s duration. Participants were asked to respond to the semantics of the colour word (colour version) or the shape of the geometric form (shape version) as fast and as accurately as possible. Immediately after the participant’s response (without any time delay), the next trial started automatically. There was only a break in Stroop tasks between the noise scenarios, when no sound was played back. The participants did the Stroop task self-paced, which resulted in a different number of trials per participant and noise scenario, depending on how fast they worked on the tasks.

The sequence of the two Stroop versions was randomized for each noise scenario, as was the sequence of the noise scenarios. After each noise scenario, participants answered the following question, which was adapted from the ICBEN noise annoyance question [3,61]: “What number from 0 to 10 represents best how much you were bothered, disturbed, or annoyed by the sound?” The participants gave their rating by means of a slider in the test program on the unipolar numerical ICBEN 11-point scale. As the spacing of the 11-point scale is equal (and thus interval-scaled), it allows treating the data as continuous in statistical analyses, even though by definition the scale is ordinal [3]. This is supported by literature, given that the ordinal variable has five or more categories [62,63,64].

After a break of 30 s the next noise scenario started. The total experiment lasted approximately 50 min, with the actual unfocussed listening test taking around 35 min.

### 3.3. Participants

The participants were mostly recruited within Empa, via internal online advertisement or direct verbal recruitment. Twenty-four persons (11 females and 13 males), aged between 19 and 63 years (median of 28.5 years), participated in experiment 1. This number of participants lies well within the range of 16–32 participants proposed in [55] to obtain reliable experimental results. All participants fulfilled the requirements for participation (self-reported normal hearing, self-reported normal or corrected-to-normal vision, not colour blind, legal age and feeling well, see above). Written consent for participation was collected from all participants. 

### 3.4. Data Analysis

Annoyance: In total 168 annoyance ratings were obtained (i.e., 24 participants × 7 road traffic noise scenarios).

Performance: Task completion was self-paced, i.e., each participant had an individual pace in completing the tasks. This resulted in different amounts of worked-out trials per noise scenario and participant. On average, 208 trials in the Stroop task were worked-out, ranging from 85–265 trials per participant and traffic noise scenario, meaning that the slowest participant completed 82 trials during one specific noise scenario, and the fastest participant 262 trials during one specific noise scenario. In sum, a total of 34,911 individual responses (trials) were available and processed as follows.

Reaction times (RTs; in ms): Each trial not correctly worked-out counted as an error. As usual in analysis of RTs, error trials were removed from the data set, as cognitive mechanisms might have been different from those involved in successful task processing. In a second step, long RTs (exceeding 2 standard deviations of mean overall RTs of the experiment, corresponding to RTs > 1771 ms) were removed, as again other mechanisms might have played a role (e.g., the participant re-reading the instructions on the task or accidentally pressing a response key). In total, 3000 individual responses (trials) (9.1%) were removed. In a last step, the remaining 31,911 individual responses were averaged per participant and road traffic noise scenario separately for congruent and for incongruent trials to obtain mean RTs (data set with a total of 336 entries).

Error rate (ER; in %): In a first step, individual colour and shape task versions/variants (cf. Section 2.2) per participant with too high rates of wrong answers (namely, ER > 10%) were removed, as these tasks were likely misunderstood by the participants (e.g., answering the colour instead of the required semantics of the word). In total, 3,410 trials (9.8%) were thus removed. The remaining 31,501 individual trials were again averaged per participant and noise scenario separately for congruent and incongruent trials to obtain the mean ERs (data set with a total of 336 entries).

The data was statistically analysed, separately for annoyance on the one hand, and RT and ER as measures of cognitive performance on the other hand. To that aim, linear mixed-effects models were established (see, e.g., [65]). These models allow separating fixed effects (here, the variables RQT and QTD, which were correlated with the other indicators, cf. Section 3.1) and random effects (the participants, modelled with a simple random intercept: one for each participant). Further, the playback number (i.e., the serial position with which the noise scenarios had been played) was included to test for order effects [66]. The statistical analysis was done with IBM SPSS Version 25 using the procedure MIXED.

### 3.5. Results

#### 3.5.1. Annoyance

Table 2 shows the correlations (Spearman’s rank correlation coefficient *r*_s_ [60] and Pearson’s *r*, the latter assuming a linear relation) of the annoyance ratings with the continuous indicators for the temporal pattern. Both correlation analyses reveal the same insights, although correlation with Spearman’s *r*_s_ is less strong than with Pearson’s *r*. Annoyance increased with increasing *N* (more events) and CMT (i.e., longer noise breaks, indicating irregular distribution of the events), but decreased with increasing RQT (longer total quiet time), *IR* (increasingly dominant, here meaning less, single events) and *L*_AF,max_ (louder, here meaning less, events). As the acoustical indicators are closely correlated to either CMT or QTD (cf. Table A1), the following account focusses on RQT and QTD. As Table 2 reveals, the correlations are rather moderate. One reason for this is that the correlation analysis was performed for the individual annoyance data (168 ratings: cf. Section 3.4) without accounting for individual differences between participants’ ratings. This shortcoming is overcome by the subsequent hierarchical mixed-effects models, where the participants are modelled with a random intercept.

**Figure 6:**
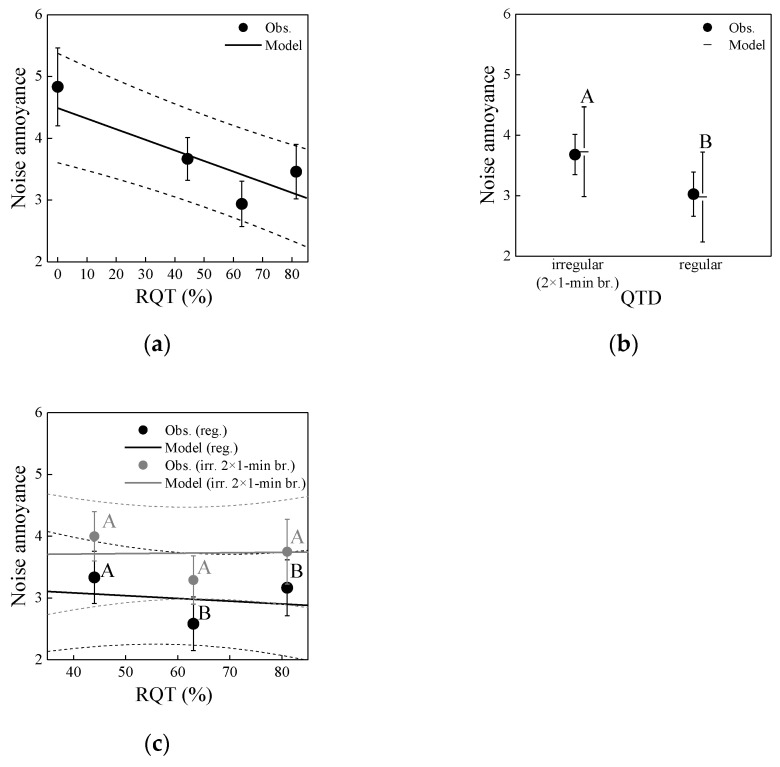
Noise annoyance as a function of (**a**) relative quiet time (RQT), (**b**) quiet time distribution (QTD) and (**c**) both RQT and QTD as found in experiment 1. Circles represent mean observed values (Obs.) with standard error bars, and lines the corresponding mixed-effects models with 95% confidence intervals, in (**b**) as horizontal lines with confidence intervals. In (**b**,**c**), significant differences between estimated marginal means (*p* < 0.05; pairwise comparisons with Bonferroni correction) of regular and irregular QTD are indicated by differing letters.

Figure 6 shows the association of annoyance with RQT and QTD. RQT increasing from 0% to 44–81% was associated with decreased annoyance. QTD was linked with annoyance as well, with regular breaks being less annoying than irregular breaks. An interaction between RQT and QTD was not observable (Figure 6c). Besides, annoyance increased with playback number increasing from 1–7 (not shown). This simple order effect was expected and observed in other studies by the same authors (e.g., [21,45]), indicating that the participants got increasingly annoyed by the road traffic noise scenarios over time.

Linear mixed-effects modelling analysis confirmed these observations and significant differences between regular and irregular QTD (cf. Figure 6b,c). Here, two models are reported, which either relate annoyance to RQT (model M_RQT_) or to QTD (model M_QDT_). The first model, M_RQT_, reveals the dependence of annoyance on the continuous variables RQT and playback number (PN). This model takes into account all noise scenarios, S0–S6.

*Annoy_k_* = *μ* + *β*_1_ × RQT + *β*_2_ × PN*_k_* + *u_k_* + *ε_k_*. (5)

In Equation (5), *Annoy* is the dependent variable annoyance, *μ* denotes the overall grand mean, *β*_1_ and *β*_2_ are regression coefficients for the continuous variables RQT and PN, respectively, of the seven scenarios (S0–S6), *u* is the participants’ random intercept (*k* = 1–24), and the error term *ε* is the random deviation between observed and expected values of *Annoy*. Table 3 gives the model coefficients. The model M_RQT_ shows that annoyance significantly decreases by 1.4 units on the 11-point scale when RQT increases from 0–81% (cf. Figure 6a), and significantly increases by 1.4 units with a playback number increase from 1–7 (incidentally a very similar increase as for RQT increasing from 0–81%).

The second model, M_QDT_, reveals how annoyance is linked to QTD. In this model, only six scenarios, S1–S6, are taken into account, since no level of QTD is applicable for S0 with RQT of 0% (cf. Table 1). In the absence of S0, RQT is not linked to annoyance (*p* > 0.8; also obvious in Figure 6c). Also, there was no significant interaction between RQT and QTD (*p* > 0.7; cf. Figure 6c). Model M_QDT_ therefore reduces to

*Annoy_ik_* = *μ* + *τ*_QTD,*i*_ + *β* × PN*_ik_* + *u_k_* + *ε_ik_*. (6)

In Equation (6), *τ*_QTD_ is the categorical variable QTD (2 levels: *i* = 1, 2 for regular and irregular) of the six scenarios (S1–S6), and the other variables have the same notation as in Equation (5). Table 4 gives the model coefficients. According to model M_QTD_, annoyance is significantly higher for longer, irregular than for shorter, regular breaks, but the difference of ~0.7 points on the 11-point scale is moderate (cf. Figure 6b). Further, annoyance significantly increases with playback number (as in above model M_RQT_).

#### 3.5.2. Cognitive Performance

Performance data was first checked for the Stroop effect with a simple model considering congruency as the sole fixed effect. In fact, the Stroop effect was found for both, RTs and ERs: Overall, the effect of congruency was highly significant for RTs (*p* < 0.001), with incongruent trials (mean RT = 682 ms; standard deviation *SD* = 148 ms) being answered 31 ms (or 5%) slower than congruent trials (mean RT = 652 ms, *SD* = 138 ms), as usual in the Stroop paradigm. Furthermore, the Stroop effect was also found for ERs (*p* < 0.05), with more errors been made in incongruent trials (mean ER = 2.4%, SD = 2.6%) than in congruent trials (mean ER = 2.0%, SD = 2.2%). Consequently, the effects of the different road traffic noise scenarios on RTs and ERs were analysed separately for congruent and incongruent trials in the following.

**Figure 7:**
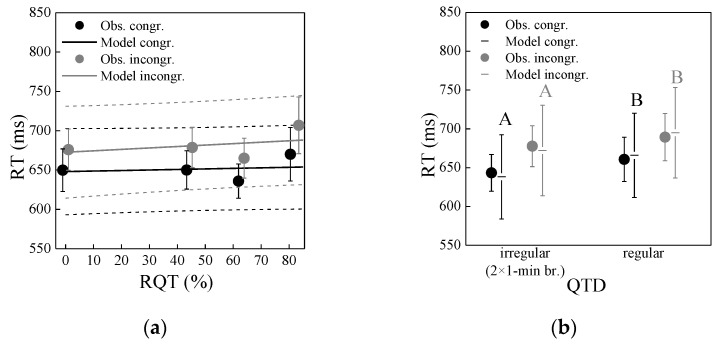
Reaction time (RT) as a function of (**a**) relative quiet time (RQT) and (**b**) quiet time distribution (QTD), for the congruent and incongruent items of the Stroop task in experiment 1. Circles represent mean observed values (Obs.) with standard error bars, and lines the corresponding mixed-effects models with 95% confidence intervals, in (**b**) as horizontal lines with confidence intervals. In (**b**), significant differences between estimated marginal means (*p* < 0.05; pairwise comparisons with Bonferroni correction, performed separately for the congruent and incongruent items) of regular and irregular QTD are indicated by differing letters.

RT: Figure 7 shows the association of RT with RQT and QTD, separately for congruent and incongruent trials in the Stroop task. RT was not linked to RQT, except that it tended to be somewhat longer for the longest RQT (81%) than the other RQTs (0–63%) (Figure 7a). RT, however, was linked to QTD, being somewhat longer for regular than irregular breaks (Figure 7b). Congruent and incongruent stimuli were affected similarly strong. Besides, RT decreased with increasing playback number (not shown) as participants got quicker with answering the trials of the Stroop task over time, indicating that they got increasingly practiced.

Linear mixed-effects model analysis again confirmed these observations and significant differences between regular and irregular QTD (cf. Figure 7b). It revealed that RT was not significantly associated with RQT for incongruent (*p* = 0.29) and congruent trials (*p* = 0.65) (cf. Figure 7a), but with QTD (*p*’s < 0.05; Figure 7b) and playback number (*p*’s < 0.001) for both incongruent and congruent trials (details not shown). While the effect of QTD was significant, it was quite small (less than 30 ms compared to overall ~650 ms RTs on average, corresponding to a relative change of less than 5%; cf. Figure 7b). RT decreased by some 140 and 130 ms for incongruent and congruent trials, respectively, with playback number increasing from 1–7.

ER: In both incongruent and congruent trials, ER varied neither with RQT nor with QTD nor with playback number (not shown), as also confirmed by mixed-effects model analysis (*p*’s > 0.30 for RQT, *p*’s > 0.26 for QTD, *p*’s > 0.23 for playback number).

## 4. Experiment 2

In experiment 2, the effects of QTD were explored in more detail. A new sample of volunteers was recruited; no one participated in both experiments.

### 4.1. Audio Processing and Resulting Road Traffic Noise Scenarios

Three road traffic noise scenarios (WAVE PCM format) were again prepared in MATLAB Version 2019a (The MathWorks, Inc., Natick, MA), in the same way and from the same recordings as in experiment 1. Furthermore, participants were also exposed to the same constant background sound at an *L*_Aeq_ of 30 dB(A) (Section 3.1). Each of the three noise scenarios was 10 min long. For training, the same two 30 s long noise scenarios as in experiment 1 were used.

**Figure 8:**
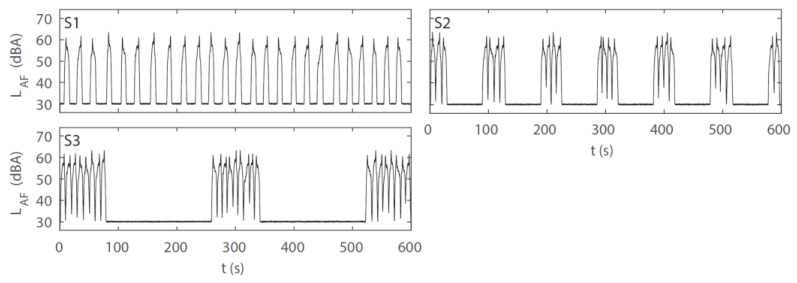
A-weighted and FAST-time weighted level-time histories (*L*_AF_) of the road traffic noise scenarios in experiment 2. S1–S3 refer to noise scenario 1–3 (cf. Table 5).

**Figure 9:**
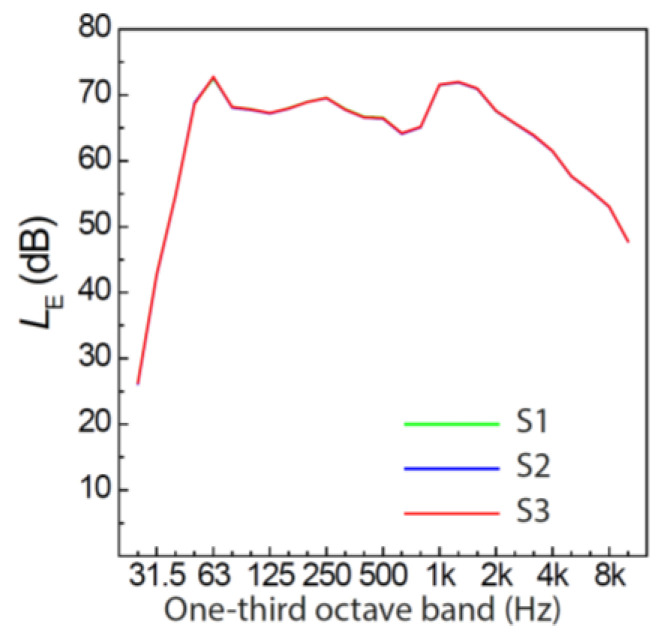
One-third octave spectra of the road traffic noise scenarios in experiment 2. S1–S3 refer to noise scenario 1–3 (cf. Table 5). Note that the three spectra are identical because the same car pass-by events were used to generate the three scenarios.

The three road traffic noise scenarios had the same RQT and *L*_AF,max_ of the individual car pass-by events, but differed with respect to QTD. Three levels of QTD were used: regular quiet periods, a combination of short quiet periods and six 1-min quiet periods, or two 3-min quiet periods (“irregular”). Each noise scenario contained 25 car pass-by events. The scenarios had an *L*_Aeq_ of 51 dB(A) at the window (measured 50 cm away from and in front of the loudspeaker) and of 41.5 dB(A) at participant’s ear level at the desk. Figure 8 shows the level-time histories of the scenarios with different QTDs and resulting lengths of the noise breaks, and Figure 9 their corresponding one-third octave spectra, which were all identical because the same individual car pass-by events were used to generate the three scenarios. Table 5 presents the indicators for the resulting macro-temporal pattern of the scenarios. Here, the association of the macro-temporal pattern with annoyance and cognitive performance was mainly investigated with QTD (as CMT was closely related to QTD, cf. Section 3.1), while RQT, *N*, and *L*_AF,max_ were the same for S1–S3 and *IR* varied only little (Table 5).

### 4.2. Experimental Procedure

The procedure of experiment 2 closely followed that of experiment 1. Experiment 2 was conducted in single sessions in English. It lasted 45–50 min, with the actual unfocused listening test taking around 32 min.

### 4.3. Participants

The participants were again mostly recruited within Empa, via internal online advertisement or direct verbal recruitment. Twenty-five persons (12 females and 13 males), aged between 26 and 61 years (median of 33.0 years) participated in experiment 2. All participants fulfilled the requirements for participation (self-reported normal hearing, self-reported normal or corrected-to-normal vision, not colour blind, legal age and feeling well; cf. Section 3.2). Written consent was collected from all participants.

### 4.4. Data Analysis

Annoyance: In total, 75 annoyance ratings (25 participants × 3 traffic noise scenarios) were obtained.

Performance: Since task completion was self-paced, different amounts of worked-out trials resulted per participant and road traffic noise scenario. On average, 452 trials in the Stroop tasks were worked-out, ranging from 301–593 trials per participant and noise scenario. In total, 33,915 individual responses (trials) were available and processed analogously as in experiment 1 (Section 3.4), removing error trials as well as RTs exceeding 2 standard deviations of mean overall RTs, corresponding to RTs > 1724 ms. Thus, 2688 individual trials (8.3%) were removed for RT analysis. For ER analysis, 3153 individual trials (9.3%) of task versions/variants with too high rates of wrong answers (again, ER > 10%) were removed to ensure sufficient task understanding. The remaining 31,227 (RT) and 30,762 individual trials (ER) were then averaged per participant, noise scenario and congruency (congruent/incongruent trials) to obtain the mean RTs (in ms) and ERs (in %) (data set with a total of 150 entries).

As in experiment 1, the data was statistically analysed with linear mixed-effects models, separately for annoyance, RT and ER. As fixed effects, QTD as well as the playback number were used, and as random effects the participants (simple random intercept). The statistical analysis was again performed with IBM SPSS Version 25 using the procedure MIXED.

### 4.5. Results

#### 4.5.1. Annoyance

**Figure 10:**
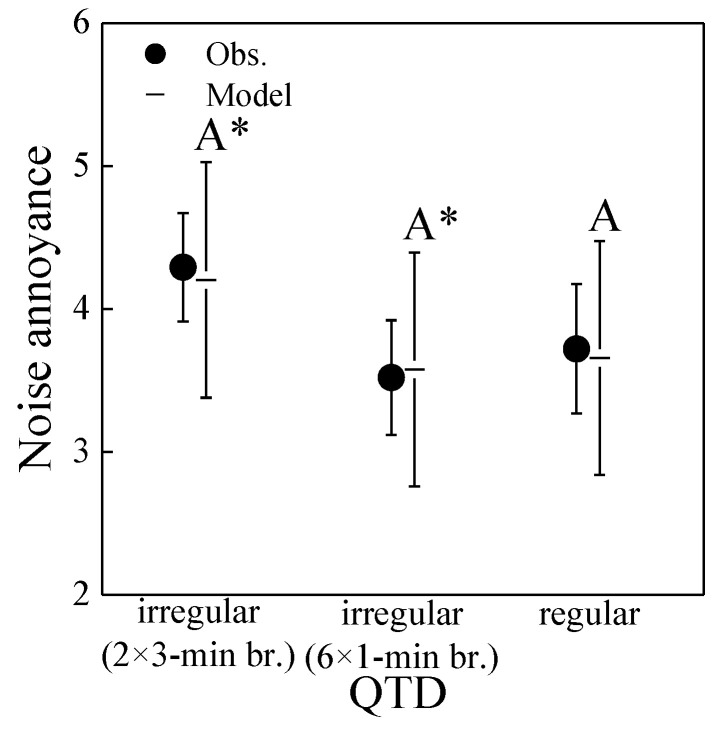
Noise annoyance as a function of quiet time distribution (QTD) in experiment 2. Circles represent mean observed values (Obs.) with standard error bars, and horizontal lines the corresponding mixed-effects model with 95% confidence intervals. Significant differences between estimated marginal means (*p* < 0.05; pairwise comparisons with Bonferroni correction) of different QTDs would be indicated by differing letters (* trend, *p* = 0.06 between irregular breaks).

Figure 10 shows the association of annoyance ratings with QTD. In line with experiment 1 (Figure 6b), annoyance was associated with QTD. The longest (3-min) breaks were somewhat more annoying than shorter breaks (irregular 1-min or even shorter, regular breaks). In contrast to experiment 1, however, the shorter irregular 1-min breaks were associated with very similar mean annoyance ratings as the regular breaks.

In line with these observations, linear mixed-effects model analysis (Table 6), using the approach of Equation (6) (model M_QDT_, but with *τ*_QTD_ with 3 levels, *i* = 1–3, for regular and irregular with 1-min or 3-min breaks), revealed that the overall association of annoyance with QTD was not significant (*p* = 0.13). In fact, only the annoyance to the 3-min and 1-min irregular breaks was in tendency different by ~0.6 units on the 11-point scale (*p* = 0.06; Figure 10). Again, playback number was significantly linked to annoyance (*p* < 0.001).

#### 4.5.2. Cognitive Performance

As in experiment 1, the performance data was first checked for the Stroop effect with a simple model considering congruency as the sole fixed effect. For both RT and ER a highly significant effect of congruency was given (*p* < 0.001), due to prolonged RTs and higher ERs during incongruent compared to congruent trials. Overall, incongruent trials (mean RT = 722 ms, *SD* = 119 ms) were answered 31 ms (or 5%) slower than congruent trials (mean RT = 691 ms, *SD* = 114 ms), and more errors were made in incongruent (mean ER = 2.0%, *SD* = 2.2%) than in congruent trials (mean ER = 1.3%, *SD* = 1.9%). Consequently, the effects on RTs and ERs were analysed separately for congruent and incongruent trials.

**Figure 11:**
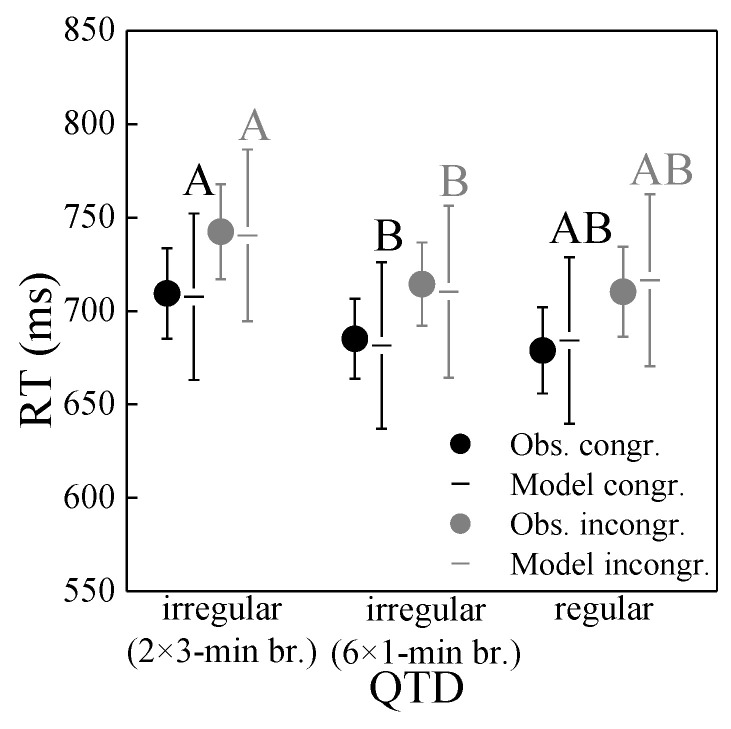
Reaction time (RT) as a function of quiet time distribution (QTD), for the congruent and incongruent trials of the Stroop task in experiment 2. Circles represent mean observed values (Obs.) with standard error bars, and horizontal lines the corresponding mixed-effects model with 95% confidence intervals. Significant differences between estimated marginal means (*p* < 0.05; pairwise comparisons with Bonferroni correction, performed separately for the congruent and incongruent Stroop tasks) of different QTDs are indicated by differing letters.

RT: Figure 11 shows the association of RTs with QTD, separately for congruent and incongruent trials in the Stroop task. RTs were linked to QTD, being longer for the longer (3-min) irregular breaks than the shorter (1-min) irregular and the regular breaks. This contrasts experiment 1, where the RTs were longer for the regular than the irregular (1-min) breaks (Figure 7). Besides, RTs decreased with increasing playback number (not shown). Congruent and incongruent trials were again affected similarly strong.

These observations and significant differences between long irregular and short irregular/regular QTD were confirmed by linear mixed-effects model analysis, which showed that RTs were significantly associated with QTD (*p* < 0.02) and playback number (*p* < 0.001) (details not shown). While the effect of QTD was significant, it was again small (around 30 ms compared to ~700 ms RTs on average, corresponding to a relative change of ~4%). RTs decreased with playback number increasing from 1–3 by some 100 and 90 ms for incongruent and congruent stimuli, respectively.

ER: In both congruent and incongruent trials, ER was neither associated with QTD nor playback number, which was also confirmed by mixed-effects model analysis (*p*’s > 0.65 for QTD, *p*’s > 0.05 for playback number).

## 5. Discussion

This study performed two unfocussed laboratory listening experiments to study how the macro-temporal pattern of different road traffic noise scenarios with rather low *L*_Aeq_ of ~45 dB(A) (experiment 1) and ~42 dB(A) (experiment 2), as might be expected in an office environment, affected short-term noise annoyance and cognitive performance in the Stroop task. A range of indicators for the macro-temporal pattern of the scenarios, including relative quiet time (RQT) and quiet time distribution (QTD), were quantified.

### 5.1. Annoyance

The experiments confirmed that quiet periods affect annoyance, revealing that annoyance ratings decreased with increasing RQT, at least up to some 60% (Figure 6). This is in line with literature [8,9,10,25,27,30,67]. Further, annoyance was linked with QTD. Shorter but more regular breaks were found to be perceived as less annoying than longer but irregular breaks of identical total duration. Similar insights as with RQT and QTD may also be obtained with the other indicators for the macro-temporal pattern (Table 2), which were closely related to either RQT or QTD (Table A1). For example, the number of events (negatively correlated with RQT) positively correlates with annoyance, which was also found for aircraft noise in [20], while *IR* (positively correlated with RQT) shows a negative correlation with annoyance, confirming the findings of [5]. In interpreting our results on *IR*, one should keep in mind that with the exception of the reference scenario S0, all scenarios were highly intermittent (cf. Figure 4 and Figure 8), with *IR* values of 74% and more. Our findings suggest that, at the same RQT (with the same number of events), the clustering of car pass-by events after prolonged quiet times (irregular QTD), giving a more distinct temporal pattern, was more annoying to the participants than the shorter but regular events. Thus, to optimize QTD in order to minimize annoyance, providing a smooth traffic flow without too many interruptions, e.g., by reducing traffic lights, might be beneficial. In line with this thought, a laboratory study found that at high traffic densities, road traffic noise at a roundabout was perceived as less unpleasant than at crossroads with traffic lights [68]. RQT, in contrast, can only be optimized (meaning, increasing the breaks) through reduced the traffic volume (e.g., with traffic and parking restrictions and charges in cities), which also positively affects the *L*_Aeq_.

The present results on QTD contrast the conclusions of previous studies that suggest a minimal duration of one [25] or three minutes [27,28,29] for a quiet period to be valuable with respect to annoyance, and of another laboratory study that did not find the duration of quiet periods to affect annoyance [67]. Thus, while breaks between events (i.e., having certain quiet periods, here: RQT) do seem beneficial, the link of the distribution of noise breaks with annoyance was less clear, and the necessity of a minimal duration of the noise breaks could not be confirmed. However, given the relatively low sound exposure in the experiments with an *L*_Aeq_ of ~42–45 dB(A), the effects were moderate only, changing annoyance by 1.4 units on the 11-point scale for a RQT increase from 0–81%, and 0.5–0.7 units for longer irregular compared to shorter quiet times (QTD).

Overall, the moderate association of annoyance with relatively low-level road traffic noise (*L*_Aeq_ of 42–45 dB(A)) is in line with a recent laboratory study that found the link between subjective disturbance and road traffic noise with an *L*_Aeq_ of 35–41 dB(A) to be quite weak [16].

### 5.2. Cognitive Performance

Compared to annoyance, the association of the macro-temporal pattern with cognitive performance in terms of RT and ER in the Stroop task was less clear. While RQT did not affect performance, QTD was slightly linked to RTs, but the results of experiments 1 and 2 were not clear-cut. In experiment 1, short regular breaks were found to be associated with longer RTs than short irregular breaks (Figure 7), but not in experiment 2. Here, long irregular breaks resulted in prolonged RTs (Figure 11). Yet in both experiments, the association of RTs with QTD, while significant, was weak, with small relative changes in RT of less than 5%. Further, no association of ER with the macro-temporal pattern of the noise scenarios was found. Similar results were also found in a preliminary listening experiment to this study [44], where road traffic noise neither affected RT nor ER.

This unsystematic effect pattern of the different noise scenarios on performance in the Stroop task might be due to their effect on attentional functions being comparatively smaller than their effect on noise annoyance, and because the applied experimental procedure did not allow for a more sensitive analysis of performance data. That is to say, the road traffic noise scenarios used in this experiment may have had too few salient changes (deviants) in terms of transitions from noisy to quiet periods (and back) diverting the attentional focus away from the task at hand to measure an effect on performance in the Stroop task when considering all trials worked out. However, the analysis of performance data could not be limited to those trials of the Stroop task that were performed at the time of, or shortly after, the salient changes in the road traffic noise scenarios. This was because the processing of the Stroop trials was self-paced in the present experiments, so that the relevant individual trials in the cognitive task could not be identified. In contrast, the above-mentioned laboratory study [16] found transitional phases in road traffic noise scenarios to affect reading task performance. Reading speed decreased as the sound level increased (rising front of an event) and increased again during the descending front.

Nevertheless, the typical Stroop effect was found in both experiments. That is, RTs were prolonged and ERs were increased for incongruent items, in which two dimensions of the visual stimulus did not match, compared to congruent items. This indicates that the participants seriously worked on the given cognitive task, and that our study in fact comprised unfocused listening experiments to investigate annoyance. Since performance in the Stroop task versions used here hardly changed during the different road traffic noise scenarios and, moreover, did not change systematically between the two experiments, differences in annoyance ratings can be assumed to not be moderated or even caused by performance effects (i.e., one was not annoyed because he/she could not perform well). Instead, the observed annoyance effects can be indeed attributed to the differing macro-temporal pattern of road traffic noise. In that context, it would be interesting to study the effects on noise annoyance in situations where also performance in (possible more difficult) cognitive tasks is affected by the macro-temporal pattern of road traffic noise.

### 5.3. Strengths and Limitations

A particular asset of the current study is that both, noise annoyance and cognitive performance, were mutually studied in two experiments to evaluate potential effects of road traffic noise comprehensively. While similar studies are available for background speech and music [39,40,41], studies involving road traffic noise to investigate such mutual effects are rare [16,17]. Besides, our design revealed that the associations of annoyance and performance with the acoustic characteristics (RQT or QTD) are quite different.

The study also faces certain limitations. As is generally true for laboratory studies, the ecological validity is limited due to the laboratory setting and the rather limited number of participants. Further, inferring from short-term noise annoyance in the laboratory to long-term annoyance in the field still needs to be verified ([69]), and inferring from cognitive performance tasks to long-term performance in office environments is similarly challenging.

Also some specific limitations apply. Above all, adopting the design to allow for a more sensitive analysis of performance data, specifically aiming at the transitional phases between quiet and loud periods (see above), would be beneficial. Besides, varying the *L*_Aeq_, which is a decisive factor for road traffic noise annoyance (e.g., [45,68]) would add an important dimension to the outcomes. If the *L*_Aeq_ was sufficiently high to substantially affect cognitive performance, one could also study the effect of reduced performance on (noise) annoyance. These limitations could be addressed and improved in future studies (cf. Section 5.4).

### 5.4. Outlook

Our experiment revealed that, for moderate sound exposure in an office environment, the macro-temporal pattern of road traffic noise affects annoyance. This was true although participants were not actively listening to the noise but were working on a cognitive task, and even though performance on that task was not systematically affected by the noise. Future research might test whether the association of the macro-temporal pattern of the road traffic noise scenarios with annoyance is different if participants actively listen to them (e.g., during relaxation in a mock garden environment). This could be studied in a focussed listening experiment, where only the sound to be subjectively evaluated is presented, without any cognitive task to be performed. 

Besides, follow-up experiments focusing more on the effects of road traffic noise scenarios on attentional functions might be set-up in such a way that the relevant trials in the cognitive task at the time of, or shortly after, the salient changes in the noise scenarios can be identified (i.e., non-self-paced trials or event based data logging). Then one could test more sensitively than in our experiments whether the transitions from traffic noise to quiet periods and back, and/or irregular breaks as unanticipated changes in the auditory background cause attentional capture.

In the experiments presented here, the levels were as one might well find them in an office environment. However, people are also exposed to traffic noise in street cafés, on balconies and in front gardens, where the sound levels can be significantly higher. Also there, people spend longer time periods and concentrate on certain cognitive tasks, if they have to or wish to. Consequently, further unfocussed listening experiments similar to the experiments presented here would be desirable to study the effect of macro-temporal pattern on annoyance and cognitive performance under substantially higher sound exposure (e.g., *L*_Aeq_ = 55–60 dB(A)). Such experiments could help further filling the gap in knowledge on the links between annoyance, performance and macro-temporal pattern of environmental sounds.

## 6. Conclusions

In unfocussed laboratory listening experiments, the associations of annoyance and cognitive performance with the macro-temporal pattern of relatively low-level road traffic noise situations were investigated in a mock office environment. In line with literature, annoyance decreased with increasing total duration of quiet periods. Also the distribution of the quiet times affected annoyance. Shorter but more regular breaks were found to be less annoying than longer but irregular breaks of identical total duration; a minimal necessary duration of noise breaks as proposed in literature could thus not be confirmed. Cognitive performance in an attention-based task, in contrast, did not systematically vary with the macro-temporal pattern of the situations. Thus, while the macro-temporal pattern of road traffic noise situations with moderate sound exposure seems playing a minor role for cognitive performance, it may still be important for annoyance of office staff.

## Figures and Tables

**Table 1:** Characterization of the macro-temporal pattern of the road traffic noise scenarios of experiment 1 (*N* = number of events, RQT = Relative Quiet Time, QTD = Quiet Time Distribution, *IR* = Intermittency Ratio, CMT = Centre of Mass Time, *L*_AF,max_ = maximum sound pressure level as maximum of the whole traffic noise scenario).

Scenario No.	*N*	RQT (%)	QTD	*IR* (%)	CMT	*L*_AF,max_ (dB(A))
S0	36	0.00	not applicable	30.4	Not applicable	61.4
S1	5	81.4	regular (shorter breaks)	94.4	36.7	67.9
S2	5	81.4	irregular (incl. 2 × 1-min breaks)	94.8	44.3	68.2
S3	10	62.9	regular shorter breaks)	89.5	15.5	65.2
S4	10	62.9	irregular (incl. 2 × 1-min breaks)	90.0	43.9	65.2
S5	15	44.3	regular (shorter breaks)	74.3	7.7	65.2
S6	15	44.3	irregular (incl. 2 × 1-min breaks)	76.0	60.3	65.2

**Table 2:** Correlation analysis: Spearman’s rank correlation coefficient *r*_s_ [60] and Pearson’s *r* for correlations between annoyance and the indicators for the macro-temporal pattern of the road traffic noise scenarios (cf. Section 2.3) (*N* = number of events, RQT = Relative Quiet Time, *IR* = Intermittency Ratio, CMT = Centre of Mass Time, *L*_AF,max_ = maximum sound pressure level).

Correlation	*N*	Log(*N*)	RQT (%)	*IR* (%)	CMT	*L*_AF,max_ (dB(A))
Spearman’s *r*_s_	0.14 ^†^	0.14 ^†^	–0.14 ^†^	–0.10	0.15 ^†^	–0.15 *
Pearson’s *r*	0.22 **	0.18 **	–0.20 **	–0.23 **	0.15 *	–0.16 **

^†^*p* < 0.08, * *p* < 0.05, ** *p* < 0.01.

**Table 3:** Model coefficients (Coeff.), 95% confidence intervals (CI) and probability values (*p*) of the linear mixed-effects model M_RQT_ for annoyance (parameters and symbols: Equation (5)).

Parameter	Symbol	Coeff.	95% CI of Coeff.	*p*
Intercept	*μ*	3.581	[2.558; 4.604]	<0.001
RQT (%)	*β*_1_	–0.017	[–0.027; –0.008]	<0.001
Playback No. (PN)	*β*_2_	0.227	[0.105; 0.349]	<0.001
Random effect variance	*u* ^2^ *_k_*	2.559	[2.028; 3.229]	<0.001
Residual variance (intercept)	*ε* ^2^ *_k_*	2.734	[1.419; 5.268]	0.003

**Table 4:** Model coefficients (Coeff.), 95% confidence intervals (CI) and probability values (*p*) of the linear mixed-effects model M_QTD_ for annoyance. (Parameters and symbols: Equation (6)).

Parameter	Symbol	Coeff.	95% CI of Coeff.	*p*
Intercept	*μ*	2.181	[1.276; 3.086]	<0.001
QTD	*τ*_QTD,*i*=irreg (2 × 1-min)_	0.747	[0.253; 1.242]	<0.005
	*τ*_QTD,*i*=reg_	0 ^a^		
Playback No. (PN)	*β*	0.200	[0.073; 0.327]	<0.005
Random effect variance	*u* ^2^ *_k_*	2.211	[1.713; 2.854]	<0.001
Residual variance (intercept)	*ε* ^2^ *_ik_*	2.417	[1.240; 4.713]	0.003

^a^ Reference.

**Table 5:** Characterization of the macro-temporal pattern of the road traffic noise scenarios of experiment 2 (*N* = number of events, RQT = Relative Quiet Time, QTD = Quiet Time Distribution, *IR* = Intermittency Ratio, CMT = Centre of Mass Time, *L*_AF,max_ = maximum sound pressure level as maximum of the whole traffic noise scenario).

Scenario No.	*N*	RQT (%)	QTD	*IR* (%)	CMT	*L*_AF,max_ (dB(A))
S1	25	58.3	regular	82.4	13.7	63.4
S2	25	58.3	irregular (incl. 6 × 1-min breaks)	83.7	61.7	63.4
S3	25	58.3	irregular (incl. 2 × 3-min breaks)	83.2	185.1	63.4

**Table 6:** Model coefficients (Coeff.), 95% confidence intervals (CI) and probability values (*p*) of the linear mixed-effects model M_QDT_ for annoyance in experiment 2. The parameters and symbols are explained in Equation (6) of experiment 1 (but with *τ*_QTD_ with 3 levels).

Parameter	Symbol	Coeff.	95% CI of Coeff.	*p*
Intercept	*μ*	2.442	[1.378; 3.505]	<0.001
QTD	*τ*_QTD, *i*=irreg (2 × 3-min)_	0.546	[−0.113; 1.205]	=0.10
	*τ*_QTD, *i*=irreg (6 × 1-min)_	−0.079	[−0.732; 0.573]	=0.81
	*τ*_QTD, *i*=regular_	0 ^a^		
Playback No. (PN)	*γ*	0.603	[0.272; 0.934]	<0.001
Random effect variance	*u* ^2^ *_k_*	1.299	[0.863; 1.957]	<0.001
Residual variance (intercept)	*ε* ^2^ *_ik_*	2.761	[1.423; 5.356]	0.003

^a^ Reference.

## Data Availability

Not applicable.

## Appendix A

**Table A1:** Correlation analysis: Scatterplots and Spearman’s rank correlation coefficient (*r*_s_) [60] of indicators for the macro-temporal pattern of the road traffic noise scenarios S0–S6 of experiment 1.

Indicators	N	RQT (%)	*IR* (%)	CMT	*L*_AF,max_ (dB(A))
N		−1.000 **	−0.973 **	−0.120	−0.911 **
RQT (%)			0.973 **	0.120	0.911 **
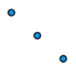
*IR* (%)				0.371	0.906 **
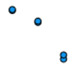
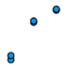
CMT					0.270
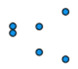
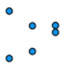
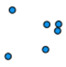
*L*_AF,max_ (dB(A))					
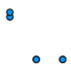
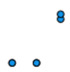
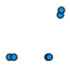
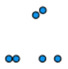

** *p* < 0.01.
